# Immediate implant placement with socket shield technique in the maxilla: a prospective case series evaluation at 1-year follow-up

**DOI:** 10.1186/s13005-022-00324-3

**Published:** 2022-06-10

**Authors:** Rola Muhammed Shadid

**Affiliations:** Department of Prosthodontics, Faculty of Dentistry, Arab American University, Jenin, Palestinian Territory, P. Box: 240, Jenin, Israel

**Keywords:** Dental implantation, Success rate, Osseointegration, Socket shield, Case series

## Abstract

**Background:**

The aims of this case series were to investigate the clinical, radiographic, implant success, complication incidence, esthetic, and patient-reported outcomes of 10 immediately placed implants associated with the socket shield technique at 12 months post-loading and to assess the ridge width changes that occurred at 8 months following implant placement.

**Methods:**

A total of 10 patients received 10 socket shield immediate implants (MegaGen AnyRidge). At 8 months postimplantation, casts were made to assess the ridge width changes by measuring the ridge width at the implant sites and comparing them with the corresponding measurements at the contralateral tooth site. At 12 months post-loading, clinical indices, marginal bone loss, pink esthetic score, and patient-assessed outcomes were evaluated. The mean, standard deviation and median were calculated for all continuous variables.

**Results:**

All implants demonstrated a 100% success rate, while 2 implants presented with external shield exposure that was managed successfully. The mean marginal bone loss was 0.08 ± 0.14 mm mesially and 0.21 ± 0.23 mm distally. Esthetic evaluation yielded an average modified pink esthetic score of 8.65. A mean gain of 0.17 mm in the facial-palatal ridge width was recorded at 8 months postimplantation.

**Conclusions:**

The socket shield technique enhanced the functional and esthetic results by preserving the alveolar bone and peri-implant soft tissues. However, this is a sensitive technique and still needs more robust evidence before it can be recommended for everyday clinical practice.

## Background

Tooth extraction is usually followed by vertical and horizontal alveolar ridge resorption [1, 2], especially on the buccal side [1]. This is highly expected because once the tooth is extracted, the alveolar ridge loses one of the main vascular supplies to the facial plate, which is the periodontal ligament [3]. In addition, the facial plate thickness in the anterior maxilla has been reported to be 1 mm or less for nearly 90 % of patients, making it more prone to surgical trauma and resorption [3, 4]. Furthermore, this 1-mm facial plate is composed mainly of cortical bone without any vascular supply from the endosseous marrow [3]. This ridge resorption has a negative subsequent impact on the implant position and on the emergence profile of the implant restoration [5], leading to aesthetic and biological complications, mainly in the maxillary anterior region. Consequently, every effort has been made to preserve or limit the physiological ridge remodeling that occurs following tooth extraction, including socket preservation techniques [6, 7] and bone augmentation using different bone materials and different membranes [8, 9].

When accompanied by immediate implant placement, numerous recommendations and techniques have been introduced for the same purpose of preserving the peri-implant hard and soft tissues. These include atraumatic tooth extraction, meticulous case selection [10], flapless implant placement [11], ideal 3-D implant positioning [11], filling the jumping gap and the area up to the gingival margin level with bone substitute (dual-zone) [12], connective tissue grafting at the time of immediate implantation [13], immediate provisionalization [14], and the use of implants with platform-switching designs [15]. However, none of those procedures could prevent the physiological bone remodeling that occurs postextraction since the main causes of the main vascular supply loss and the thin buccal bundle bone are still present [16].

As a result, a previously introduced technique of root submergence that was first used in completely edentulous ridges to maintain the denture supporting area was reevaluated [17]. Then, this technique was utilized to preserve the alveolar ridges under pontics of fixed partial dentures [18]. Based on the same concept of maintaining the periodontium of the periodontal ligament, cementum and bundle bone, Hürzeler and colleagues [19] in 2010 introduced the “socket shield” technique”. This technique involves maintaining the facial segment of a root that is intended to be extracted and immediately replaced with an implant by decoronating the tooth, sectioning the root mesiodistally, and then removing the palatal segment with the apex while maintaining the facial segment. The implant is then placed palatal to the shield [19].

Although several systematic reviews [20–23], human randomized clinical studies [24–26], and case series [15, 27, 28] have assessed the socket shield technique, more scientific evidence is still required for this method to be recommended for use in everyday clinical practice. Thus, the objective of this prospective case series of 10 participants was to investigate the clinical, radiographic, implant success, complication incidence, esthetic, and patient-reported outcomes of ten immediately placed implants with the socket shield technique at the 12-month follow-up appointment after definitive crown delivery. The facial-palatal ridge dimensional changes that occurred at 8 months following implant placement were also assessed. Additionally, the influence of probable confounders, such as gingival biotype, facial plate thickness, and healing pattern, on the abovementioned variables was evaluated.

## Materials and methods

This prospective case series study was conducted between June 2019 and June 2020 and included participants who were scheduled at the Faculty of Dentistry, Arab American University, Palestinian Territory. The study was approved by the Arab American University scientific research council (SRC-17/18-10) and performed according to the ethical principles of the Declaration of Helsinki of 1975, as revised in 2013.

### Patient selection

The inclusion criteria were any patient requiring at least one immediate implant in the maxilla from the right first premolar to the left first premolar for a nonmobile tooth between two natural teeth, with an existing contralateral tooth; being at least 20 years old; and being able to sign an informed consent form. The exclusion criteria were general contraindications to implant surgery, radiotherapy in the head or neck area, chemotherapy for malignancy in the previous 5 years, uncontrolled diabetes, severe psychiatric disease, patients taking intravenous bisphosphonates, smoker of more than 9 cigarettes a day, pregnant or lactating women, severe parafunctional activity, vertical root fracture including the facial root or horizontal fracture apical to facial bone crest, mobility of grade two or more, moderate or severe periodontitis with more than three millimeters attachment loss, ankylosed tooth, class II or III extraction sockets, and not attending the follow-up appointments. In addition, acute infection or fistula in the site planned for implant insertion was an exclusion criterion; however, chronically infected sockets [29] with small periapical lesions that comprise less than one-third of the root length were eligible to be included in the study.

All patients received thorough explanations about the provided treatment and signed a written informed consent form in which all treatment risks were explained prior to being enrolled in the study. All the surgical and prosthetic procedures were performed by one experienced dentist (R.S.), and all patients were followed for at least 1 year after definitive restoration delivery.

### Presurgical assessment

Scaling and oral hygiene instructions were given for each patient 2 weeks before the surgery. Cone beam computed tomography (CBCT) was ordered for each patient; the purpose of CBCT was to evaluate the site for the presence of intact bone plates, if there was any pathology, and to assess the sagittal position of the root. All patients received a single dose of prophylactic antibiotics 1 hour prior to the intervention (2 g of amoxicillin or 600 mg of clindamycin, if allergic to penicillin). Additionally, patients rinsed with chlorhexidine mouthwash 0.2% for 1 minute prior to the intervention.

### Clinical procedure

**Fig. 1:**
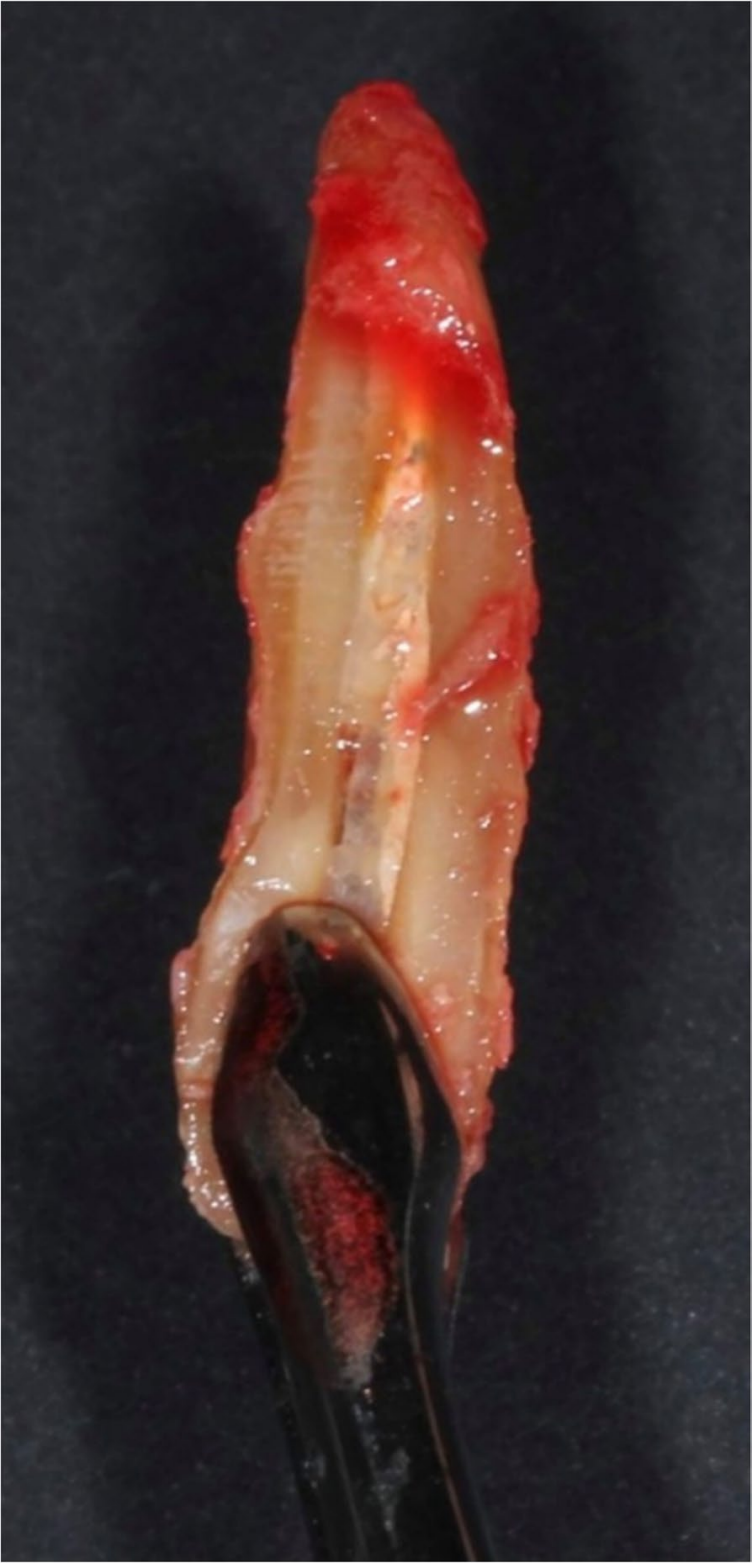
The apex was removed with the palatal portion of the root for SS preparation

The surgical procedure comprised immediate implant placement with the socket shield technique. After local anesthesia administration, the involved tooth was decoronated up to the gingival margin with a high-speed diamond chamfer bur under water irrigation. Then, the root canal was widened with successively increasing diameter Gates Glidden burs up to the apical region to remove all canal contents. To section the root mesiodistally, a long shank high-speed root resection bur (Komet Dental, Germany) was inserted into the same path created by the Gates Glidden burs until the root was sectioned completely. If possible, the apical portion was removed with the palatal portion from the first cut (Fig. 1). A small periotome was used to luxate the palatal section toward the space created by sectioning while maintaining finger support on the facial aspect of the socket to verify if there was any movement during luxation of the palatal segment. Thorough debridement, curettage, and rinsing with sterile saline were performed to remove any residues to prevent infection. To prepare the coronal portion of the shield, a microfacial flap was raised to ensure the shield was cut at the level of the facial bone crest without traumatizing the gingiva. Then, a bevel was made on the coronal 2 mm portion of the shield internally with a high-speed round diamond bur. This bevel aimed to create a more prosthetic space while reducing the risk of shield exposure. A minimum thickness of 1.5 mm and a length of 6 mm were the ideal dimensions. All procedures were conducted with magnification and high illumination. If there was any fenestration, it was managed by raising a semilunar flap in the apical area with access to the defect to ensure complete removal of infected tissue. After assessing the stability of the shield, an implant osteotomy was prepared according to the manufacturer’s instructions of the implant system (MegaGen AnyRidge, MegaGen Implant Co., Ltd., South Korea), and the implant was placed with a handpiece toward the palatal wall up to 4 mm apical to the mid-facial gingival margin. This implant with an aggressive thread design and with a 5-degree Morse Taper connection made it suitable for immediate placement and for socket shield technique [30].

**Fig. 2:**
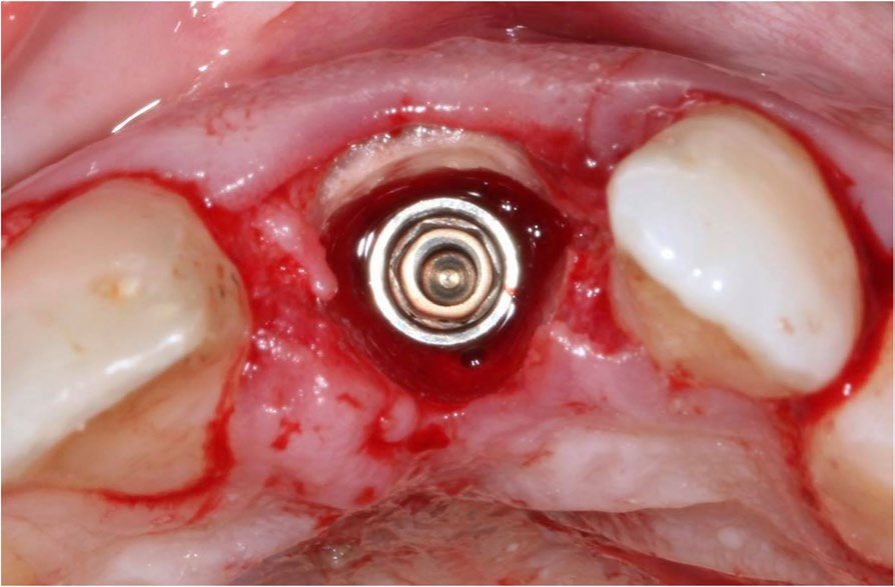
Incisal view showing implant placement palatal to the prepared facial shield; the gap was filled with mineralized allograft particles

**Fig. 3:**
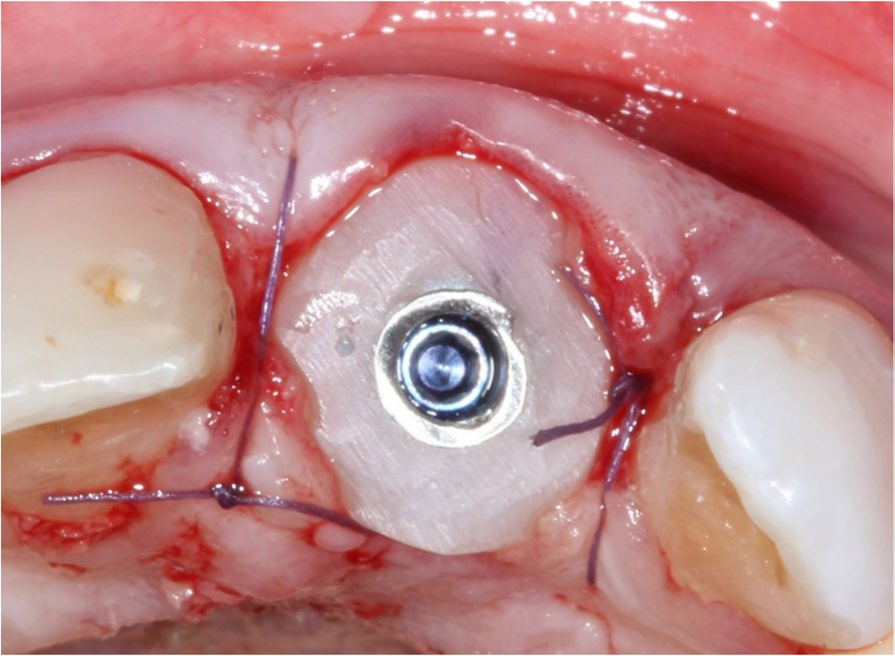
Incisal view of the customized healing abutment attached to the implant at the time of implant placement. The customized healing abutments were fabricated chairside by attaching a prefabricated titanium abutment with serrations created on its walls to retain the composite. Then, a flowable composite resin was injected to capture the soft tissue contour at the site remote from the wound to avoid contamination from the composite resin. The customized healing abutment was removed from the mouth, and a flowable composite resin was added to create a smooth transition between the abutment and the outline of the resin

Periapical radiographs were taken for each implant to verify the position and angulation of the implant; insertion torque (IT) and ISQ (implant stability quotient) values (MegaISQ; MegaGen Implant Co., Ltd) were also registered for each implant. The implant diameter was selected such that the implant would not contact the shield at the same time to be appropriate for the replaced tooth (Fig. 2). Granules of freeze-dried bone allograft (FDBA Mineross, Biohorizons IPH, Birmingham, AZ, USA) were loosely packed into the jumping distance, regardless of its size, and up to the gingival margin. Any implant with IT≥25 N/cm and ISQ ≥ 65 was attached with an S-shaped customized healing abutment (Fig. 3), and any implant with less than those readings was selected for submerged healing by placing a collagen sponge on the top of bone granules and then stabilizing it with a 5/0 polyamide nylon horizontal mattress and interrupted sutures (Filapeau, PETERS, France) without attempting primary closure. For provisionalization, a resin-bonded bridge was stabilized to adjacent teeth.

### Postsurgical protocol

The patients were asked to take antibiotics (amoxicillin 500 mg three times daily for 7 days), chlorhexidine gluconate 0.2% oral rinse 2 times daily for 2 weeks [31], and nonsteroidal anti-inflammatory drugs (ibuprofen 400 mg four times daily for 3 days). Patients were also instructed to avoid brushing the area for 2 weeks. Postsurgical evaluation was performed at 1, 3, and 6 weeks to verify whether there was any complication or infection. Sutures were removed during the 2-week postoperative visits.

### Prosthetic procedure

After approximately 4 months, all patients were asked to visit the center to start with the prosthetic phase. For nonsubmerged implants, the customized healing abutment was removed, and the ISQ was measured to ensure that the implant was ready for definitive prosthesis. If the ISQ was ≥70, the definitive prosthesis’s impression was made. Submerged implants were uncovered with the punch technique without any soft tissue enhancement, and the ISQ was also assessed. All implants were ready for loading. To shape the peri-implant soft tissue, a screw-retained provisional crown was attached to the implant for approximately 4 weeks before making the final impression. Pick-up implant-level impression copings were joined to the implants with flowable composite injected into the sulcus to transfer the soft tissue emergence profile to the soft tissue cast, and then impressions were made with putty soft/light body addition silicone material (Elite HD+, Zhermack SpA, Italy). Subsequently, screw-retained zirconia crowns were joined to the implants, and the screws were torqued to 35 N/Cm using a calibrated torque wrench (MegaGen Implant Co., Ltd., South Korea). The access holes were sealed with Teflon tape and flowable bulk fill composite (Palfique Bulk Flow, Tokuyama Dental Corporation, Japan). Periapical radiographs were taken immediately after crown delivery as a postprosthetic baseline. Maintenance and follow-up appointments were scheduled every 3 months [32].

### Outcome measures

#### Clinical indices

The modified plaque index (mPI) [33], sulus bleeding index (SBI) [34], and probing depth (PD) were assessed at mesial, distal, buccal, and palatal aspects of the implant definitive crown at delivery and at 12 months post-loading. The width of the keratinized facial mucosa (WKM) was also evaluated. Nearly 25 g probing force created with a 15-mm periodontal probe (Hu-Friedly) was used to investigate the probing depth to the nearest millimeter. Based on the mean of the four obtained readings, one value was registered for each clinical index [35].

#### Radiographic evaluation

With regard to the analysis of mesial and distal crestal bone level changes from definitive crown delivery until 12 months later, the distance from the implant platform to the first visible bone-implant contact was measured using CS Imaging Software (7.0.3 Carestream, USA) on standardized digital periapical radiographs stabilized with Kerr sensor holders (Kerr Dental, USA) and taken with the parallax technique. To facilitate the process, the marginal bone coronal to the implant platform was regarded as 0.0, while a length of 0.8 mm between the tips of the implant threads was used for measurement calibration. All measurements were executed twice by one trained dentist (F.M.), and the average of both values was calculated for mesial and distal sides.

#### Implant success assessment

At 12 months after definitive crown delivery, implant success was evaluated according to the Smith and Zarb criteria [36]. If the implant exhibited mobility, constant pain, infection, peri-implant radiolucency, more than 1.5 mm vertical bone resorption in the first year of loading and > 0.2 mm annually after the first year of loading, or improper prosthetic position, the implant was considered a failure.

#### Esthetic evaluation

A modified pink esthetic score (mPES) composed of five elements was used to evaluate the peri-implant soft tissue esthetics. Mesial papilla, distal papilla, level of the peri-implant soft tissue facially, curvature of the peri-implant soft tissue facially, and root convexity/soft tissue color and texture, also facially, were evaluated with a record of 0, 1, or 2 assigned to each one; thereby, 10 was the maximum esthetic score and a score ≥ 6 was regarded as clinically acceptable [37]. For this purpose, digital photographs captured at 12 months after definitive crown delivery with a digital single-lens reflex camera (Canon EOS 60 D, Tokyo, Japan, 100-mm Canon macro lens with ring flash) were independently assessed by two dentists (F.M. and O.S.) using a standardized procedure.

#### Patient-reported outcomes

At 12 months post-loading, the patients were requested to reply to the following questions regarding their satisfaction with the definitive crown, the peri-implant soft tissue, and the comprehensive treatment [38].

- S1) From 0 to 10, how would you rate your satisfaction concerning the definitive crown?
- S2) From 0 to 10, how would you rate your satisfaction concerning the peri-implant soft tissue?
- S3) From 0 to 10, how would you rate your satisfaction concerning the comprehensive treatment?

#### Evaluation of the facial palatal ridge width changes

Putty/light body polyvinylsiloxane impressions (Elite HD+, Zhermack SpA, Italy) were made at 8 months after implant placement to analyze the changes in the facial palatal ridge width by using a method described by Tarnow et al. [3]. Measurements were made on type 3 gypsum (Marmodent, Siladent, Germany) casts with an electronic digital caliper of 0.01 mm resolution (Salvin Dental Specialties, USA) on both the implant site (test) and the contralateral tooth site (control) at six designated points starting from the free gingival margin and progressing toward the apical area (0, 1, 2, 3, 5 and 7 mm). All measurements were recorded three times at each point by a trained assessor (F.M.), and the average of the three values was calculated.

### Statistical analysis

Data were analyzed statistically using the Statistical Package for Social Sciences (SPSS), version 22.0. Descriptive statistics of the mean, standard deviation and median were calculated for all continuous variables. The influence of probable confounders, such as gingival biotype, facial plate thickness, and healing pattern on the abovementioned variables was evaluated using independent samples t test for normally distributed data and Mann–Whitney U test for nonnormally distributed data. The level of statistical significance was considered at *P* < .05. For changes in the facial-palatal ridge width, changes in the mesial and distal marginal bone level and for the total scores of mPES, a two-way mixed effects model of consistency was used to calculate an intraclass correlation coefficient (ICC).

## Results

**Fig. 4:**
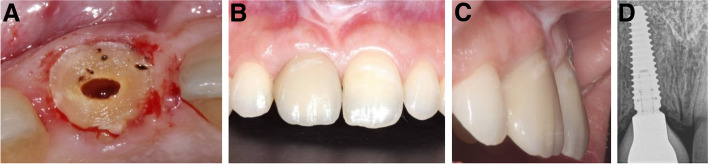
**A-D**: Photos and a radiograph of case # 1 replacing the maxillary right central incisor. **a** Preoperative incisal view; **b** frontal view of the definitive crown at 12 months postloading; **c** lateral view of the definitive crown and peri-implant soft tissue at 12 months postloading; and **d** periapical radiograph at 12 months postloading

**Fig. 5:**
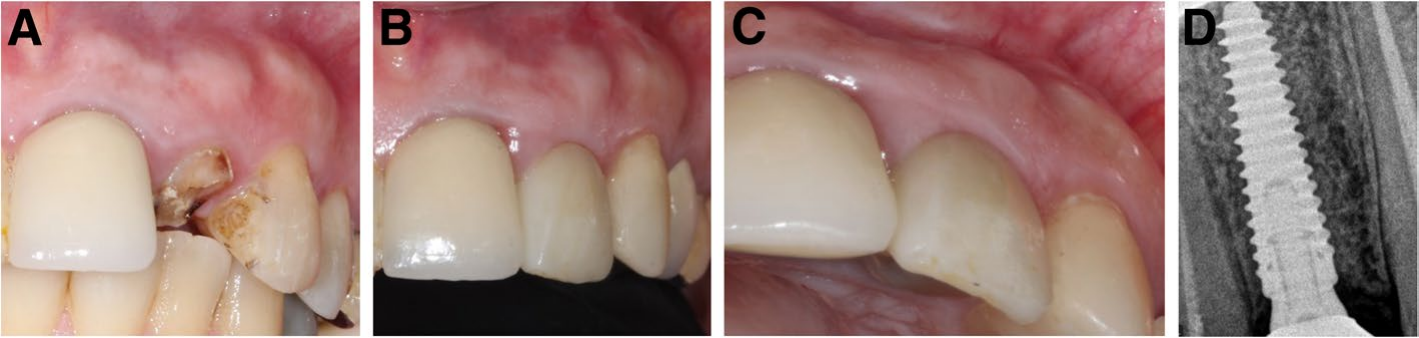
**A-D**: Photos and a radiograph of case #2 replacing the maxillary left lateral incisor. **a** Preoperative frontal view; **b** frontal view of the definitive crown at 12 months postloading; **c** incisal view of the definitive crown at 12 months postloading; and **d** periapical radiograph at 12 months postloading

**Fig. 6:**
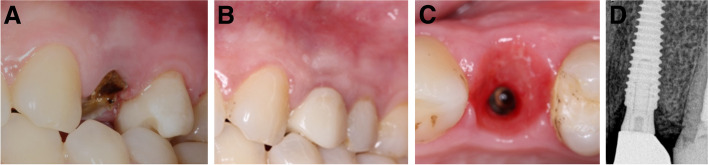
**A-D**: Photos and a radiograph of case #3 replacing the maxillary left first premolar. **a** Preoperative frontal view; **b** frontal view of the definitive crown at 12 months postloading; **c** occlusal view of peri-implant soft tissue at 12 months postloading; and **d** periapical radiograph at 12 months postloading

**Fig. 7:**
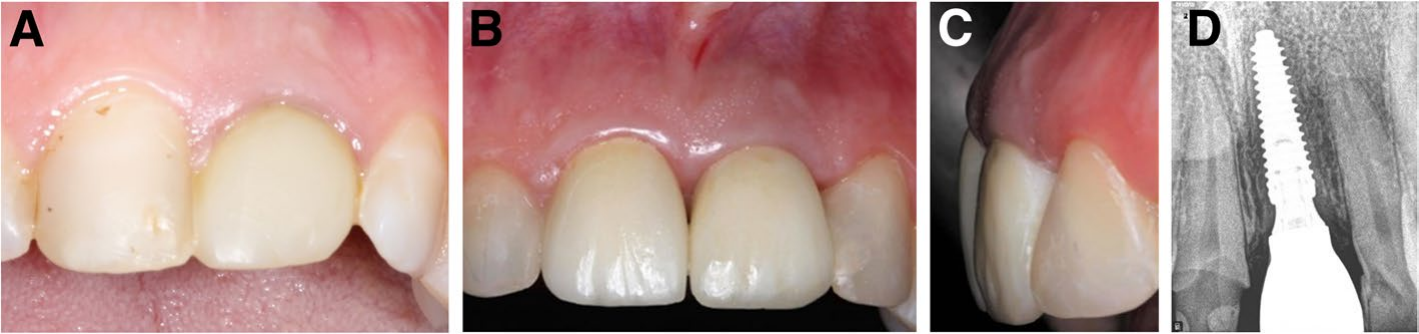
**A-D**: Photos and a radiograph of case #4 replacing the maxillary left central incisor. **a** Preoperative frontal view; **b** frontal view of the definitive crown at 12 months postloading; **c** lateral view of the definitive crown and peri-implant soft tissue at 12 months postloading; and (**d**) periapical radiograph at 12 months postloading

**Fig. 8:**
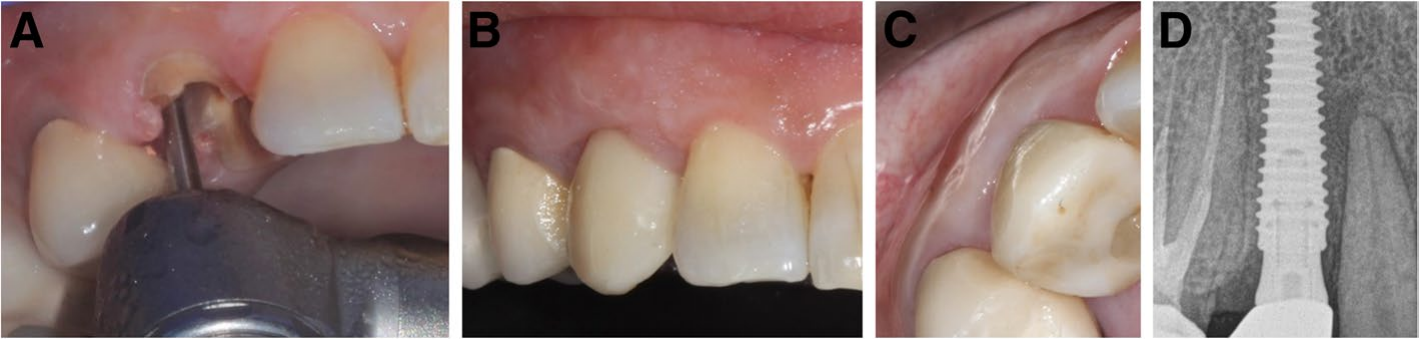
**A-D**: Photos and a radiograph of case #5 replacing the maxillary right canine. **a** Preoperative incisal view; **b** frontal view of the definitive crown at 12 months postloading; **c** incisal view of the definitive crown at 12 months postloading; and **d** periapical radiograph at follow-up postloading

**Fig. 9:**
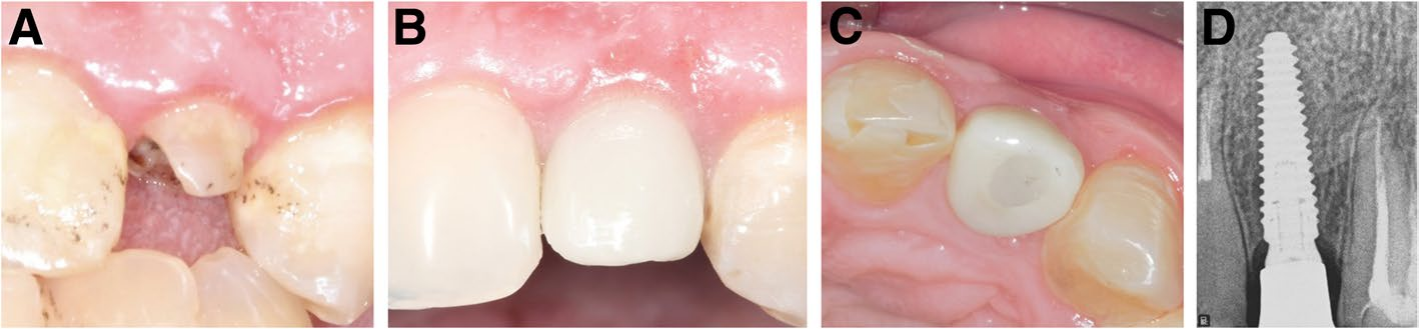
**A-D**: Photos and a radiograph of case #6 replacing the maxillary left lateral incisor. **a** Preoperative frontal view; **b** frontal view of the definitive crown at 12 months postloading; **c** incisal view of the definitive crown at 12 months postloading; and (**d**) periapical radiograph at 12 months postloading

**Fig. 10:**
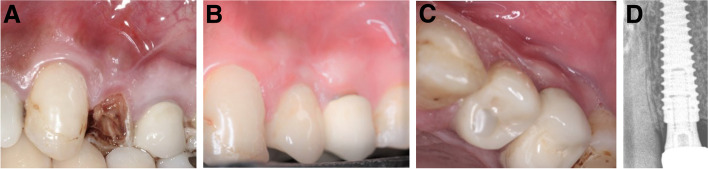
**A-D**: Photos and a radiograph of case #7 replacing the maxillary left first premolar. **a** Preoperative frontal view; **b** frontal view of the definitive crown at 12 months postloading; **c** occlusal view of the definitive crown at 12 months postloading; and **d** periapical radiograph at 12 months postloading

**Fig. 11:**
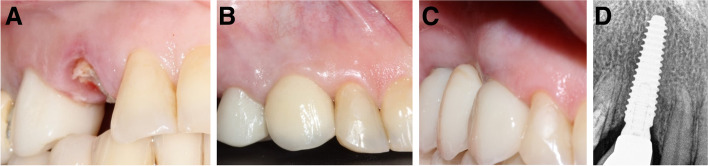
**A-D**: Photos and a radiograph of case #8 replacing the maxillary right canine. **a** Preoperative frontal view; **b** frontal view of the definitive crown at 12 months postloading; **c** lateral view taken at > 2 years after implant placement with extraction of the first premolar and insertion of FPD supported by implants in the canine and second premolar regions; and **d** periapical radiograph at 12 months postloading. *Note: The patient was told of the poor prognosis of the first premolar at the time of canine implant placement but chose to maintain it

**Fig. 12:**
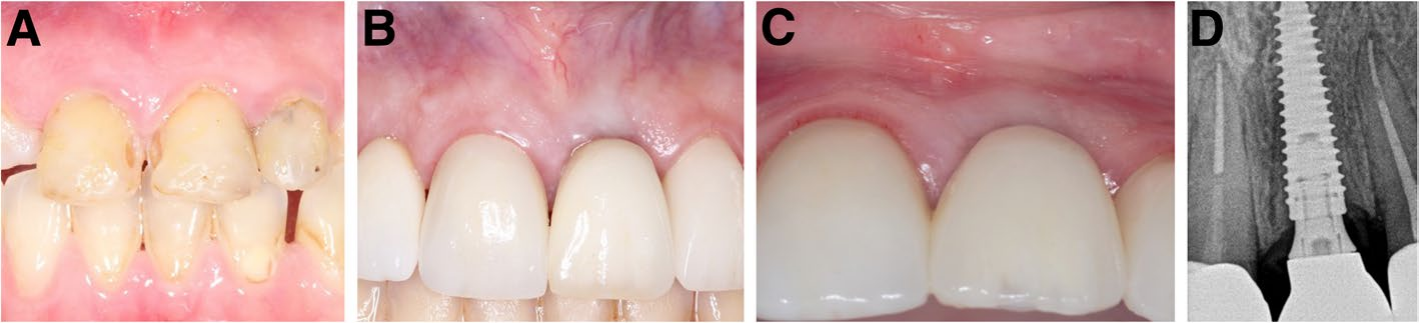
**A-D**: Photos and a radiograph of case #9 replacing the maxillary left central incisor. **a** Preoperative frontal view; **b** frontal view of the definitive crown at 12 months postloading; **c** incisal view of the definitive crown at 12 months postloading; and **d** periapical radiograph at 12 months postloading

**Fig. 13:**
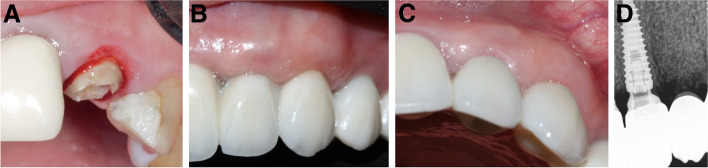
**A-D**: Photos and a radiograph of case #10 replacing the maxillary left lateral incisor. **a** Preoperative frontal view; **b** frontal view of the definitive prosthesis at 12 months postloading; **c** incisal view of the definitive prosthesis at 12 months postloading; and **d** periapical radiograph at 12 months postloading. *Note: the left central incisor and the left canine fractured during the 12-month follow-up period that led to a change in the type of restoration, from crown to fixed partial denture, supported by implants in the left central incisor, lateral incisor and first premolar areas, while the canine was transferred to the pontic shield area

Ten patients, two men and eight women with a mean age of 41 ± 12 years (range: 25–62 years), were enrolled in the study. One immediate implant was placed for each patient. The characteristics of the enrolled cases, tooth type, causes of extraction, implant dimensions, healing pattern, and definitive restoration type are shown in Table 1. The clinical pictures and radiographs of the 10 patients are presented in Figs. 4, 5, 6, 7, 8, 9, 10, 11, 12 and 13.

**Table 1:** The characteristics of the enrolled cases, tooth type, causes of extraction, implant dimensions, healing pattern, and definitive restoration

Patient No.	Age (years)	Sex	Tooth type	Extraction cause	Facial plate thickness (mm)	Gingival biotype	HP	Inserted implant (AnyRidge)	Smoker	Crown
1	25	F	Central incisor	Lack of ferrule	≥ 1	Thick	S	3.5 × 15	No	Zircon screw-retained
2	45	F	Lateral incisor	Horizontal fracture	≥ 1	Thick	S	3.5 × 11.5	No	Zircon screw-retained
3	47	F	1st premolar	Deep subgingival caries	≥ 1	Thick	NS	3.5 × 15	No	Zircon screw-retained
4	30	F	Central incisor	Lack of ferrule	< 1	Thick	NS	3.5 × 15	No	Zircon screw-retained
5	52	F	Canine	Horizontal fracture	≥ 1	Thick	S	3.5 × 15	No	Zircon screw-retained
6	35	M	Lateral incisor	Deep subgingival caries	< 1	Thick	NS	3.5 × 15	No	Zircon screw-retained
7	27	M	1st premolar	Deep subgingival caries	≥ 1	Thick	S	3.5 × 15	Yes^a^	Zircon screw-retained
8	62	F	Canine	Horizontal fracture	< 1	Thin	NS	3.5 × 15	No	Zircon screw-retained
9	38	F	Central incisor	Lack of ferrule	≥ 1	Thick	S	3.5 × 15	No	Zircon screw-retained
10	49	F	Lateral incisor	Horizontal fracture	< 1	Thick	NS	3.5 × 13	No	Zircon screw-retained

*HP* healing pattern, *S* submerged, *NS* nonsubmerged

^a^Less than 10 cigarettes per day

All patients attended all follow-up appointments. Uneventful healing without any postoperative complications occurred for all implants except 2 implants that presented with an external shield exposure. The two external shield exposures where the coronal portion of the shield perforated the soft tissue were managed by just reducing the exposed part with a high-speed diamond bur, and both healed well with soft tissue coverage.

Until the last follow-up appointment (12 months after definitive crown delivery), no unfavorable events were recorded by the patients. The peri-implant soft tissue revealed low plaque and sulcus bleeding indices, and probing depths were within normal limits (2.4 ± 0.38 mm) (Table 2). An adequate amount of facial keratinized mucosal width of 3–6 mm was recorded.

**Table 2:** Results of ridge width changes, peri-implant soft tissue health, and peri-implant marginal bone loss

Patient No.	Mean facial-palatal change (mm)	Clinical indices			MBL mesial (mm)	MBL distal (mm)	Mean of mesial and distal
		SBI	mPI	PD			
1	0.71	0.0	0.0	2.0	0.2	0.4	0.3
2	0.87	0.0	0.0	2.0	0.0	0.0	0.0
3	0.26	0.5	0.33	3.0	0.3	0.0	0.15
4	−0.12	0.0	0.0	2.33	0.0	0.45	0.23
5	0.97	0.0	0.0	2.33	0.0	0.0	0.0
6	−0.35	0.5	0.17	2.66	0.0	0.0	0.0
7	−0.64	0.33	0.33	3.0	0.0	0.35	0.17
8	0.32	0.0	0.0	2.0	0.0	0.3	0.15
9	−0.69	0.33	0.0	2.33	0.0	0.6	0.3
10	0.38	0.0	0.0	2.33	0.34	0.0	0.17
Mean	0.17	0.17	0.08	2.4	0.08	0.21	0.15
Median	0.26	0.0	0.0	2.33	0.0	0.15	0.16

*SBI* sulcus bleeding index, *mPI* modified plaque index, *PD* pocket depth, *MBL* marginal bone loss

Radiographic assessment of the 10 implants did not display any sign of continuous radiolucency during the whole follow-up interval. The mean loss of the marginal bone level equaled 0.08 ± 0.14 mm at the mesial aspect and 0.21 ± 0.23 mm at the distal aspect of the implants (Table 2). Analysis of ridge width changes in the facial-palatal direction at 8 months postimplantation showed a mean gain in facial-palatal ridge contour of 0.17 mm (Table 2).

Concerning the esthetic results, evaluation of the modified pink esthetic score from digital photographs revealed a mean score of 8.65 (Table 3). In addition, all patients demonstrated excellent contentment with the overall treatment, with the definitive crown, and with the peri-implant soft tissue outcomes (Table 3).

**Table 3:** Results of modified pink esthetic scores, patient-assessed outcomes, and complications

Patient No.	mPES	Patient-reported outcomes			Complications
		S1	S2	S3	
1	10	9	10	10	Nil
2	9.5	10	10	8	Nil
3	9	10	10	10	Nil
4	9	10	10	10	Nil
5	8.5	10	10	10	Nil
6	8	10	10	10	Nil
7	8.5	10	8	9	EX
8	9	10	10	10	Nil
9	7	10	10	10	EX
10	8	10	10	10	Nil
Mean	8.65	9.9	9.8	9.7	
Median	8.75	10	10	10	

mPES: total modified pink esthetic score; S1: patient satisfaction of definitive restoration; S2: patient satisfaction of peri-implant soft tissue; S3: patient satisfaction of overall treatment. *EX* external shield exposure

Regarding implant success evaluation, all implants demonstrated successful osseointegration, with ISQ values increasing up to ≥70 in the 4-month healing period. Until the last follow-up visit ≥12 months after loading, all implants were solid and functional without any discomfort or inflammation. Radiographic assessment did not display any sign of continuous radiolucency or vertical bone loss more than 1.5 mm in the first year of loading. According to the Smith and Zarb success criteria, the implant success rate was 100% at 12 months post-loading. With regard to the two cases with external shield exposure, more dimensional ridge shrinkage was recorded for the two patients, # 7 and #9 (− 0.64, − 0.69 mm); however, the mean marginal bone loss was 0.17 and 0.3 mm, respectively; the soft tissue looked healthy with 3 and 2.33 mm probing depths; and both patients were satisfied with the esthetic result and refused any connective tissue grafting.

No effect was found for gingival biotype, buccal plate thickness if ≥1 or < 1, or healing pattern if the implant submerged or not submerged on the clinical indices, peri-implant marginal bone loss, pink esthetic score, patient satisfaction, or on the facial-palatal ridge width changes (*P* > .05).

### Interassessor and intraassessor correlation

The intra-assessor ICC obtained for the change in the facial-palatal ridge width was 0.998 (95% CI: 0.997 to 0.999) and was 0.933 (95% CI: 0.754 to 0.983) for the change in marginal bone level. The interassessor ICC obtained for the total mPES scores was 0.800 (95% CI: 0.382 to 0.946). This indicated a high concurrence between the different measurements.

## Discussion

This prospective case series study was conducted to evaluate the clinical, radiographic, implant success, esthetic, patient-reported outcomes, and horizontal ridge changes of implants immediately placed with the socket shield technique at the 12-month follow-up appointment after loading. Therefore, single-tooth implants were inserted for patients requesting replacement of their teeth in the maxillary esthetic zone. The results of this case series ensured that the socket shield technique might enhance functional and esthetic outcomes by preserving the alveolar bone and peri-implant soft tissues.

The protocol that was followed for performing the socket shield technique in the present study was designed according to the most recent proposed guidelines [27]. The space between the implant and the facial shield when available was filled with mineralized allograft particles to prevent soft tissue ingrowth and to enhance the bone–implant contact [24, 27].

Radiographically, all implants showed good marginal bone stability at 12 months postloading, with an average marginal bone loss of 0.08 mm mesially and 0.21 distally. Although different follow-up times were considered, this result is in accordance with the retrospective case series of Baumer et al. [15], who demonstrated an average bone loss of 0.33 mm mesially and 0.17 mm distally for 10 socket shield immediate implants at the five-year follow-up. Furthermore, Bramanti et al. [24], in their randomized clinical study, reported an average marginal bone loss of 0.61 mm at the 3-year follow-up for 20 socket shield immediate implants.

Immediate implant placement with the socket-shield technique aims to prevent or minimize the postextraction resorption of the alveolar bone [19]. A retrospective case series study of 10 socket shield immediate implants revealed an average loss of just 0.37 mm from the facial-palatal ridge contour at the five-year follow-up, suggesting that this technique could effectively preserve the peri-implant tissue contours [15]. Another prospective case series of 15 socket shield immediate implants showed that the average collapse that occurred facially at 3 months postimplantation was 0.07 mm [28]. Although a direct comparison with those studies is not feasible due to the different measurement techniques used and due to different follow-up periods, the results of the present study are in agreement with the findings of those studies. At the 8-month follow-up, the present patient group revealed a mean gain in ridge contour of 0.17 mm. The slight volume increase might be due to unavoidable measurement error rather than an actual gain. The reason the socket shied technique resulted in this excellent maintenance of the ridge width is explained by the fact that maintenance of the facial shield periodontal ligament and the facial bundle bone could minimize the physiological bone remodeling that occurs postextraction [16].

Regarding the esthetic outcomes at 12 months postloading, assessment of the modified pink esthetic score yielded an average score of 8.65 (out of 10), with one case showing a total mPES of 7. This case is the same as that subjected to a traumatic accident leading to external shield exposure. However, an mPES ≥6 was considered to be clinically acceptable [37]. In addition, this patient had a low lip line, and she was satisfied with the esthetic result. The good average of the mPES reported in this study is supported by the results of different published studies [15, 22, 25]. A randomized controlled study showed an average PES of 12.2 after 12 months of follow-up with the socket shield group [25]. A retrospective case series of 10 implants immediately placed with the socket shield technique showed a mean pink esthetic score of 12 at the 5-year follow-up [15]. Furthermore, a recent systematic literature review and meta-analysis of clinical studies that evaluated the pink esthetic score of immediate implant placement with the socket shield technique reported a mean pink esthetic score of 12.27 [22]. This good esthetic outcome could be explained by the maintenance of the marginal bone crest around the implants and by the minimum volumetric alterations in the hard and soft tissues that occurred postimplantation.

Osseointegration and the rehabilitation of function and esthetics are vital factors for implant success; patient satisfaction should also be taken into consideration. In this study, all patients demonstrated excellent satisfaction with the overall treatment, with the definitive crown, and with the peri-implant soft tissue outcomes.

With respect to the implant success rate, the present study recorded a 100% success rate at 16 months postimplantation according to the Smith and Zarb success criteria. Although most studies in the literature reported implant survival rather than the success rate, this finding is in agreement with other studies that assessed the survival rate when the socket shield was associated with immediate implant placement [15, 24, 25]. However, a recent systematic literature review and meta-analysis [22] of 16 clinical studies comprising 599 implants demonstrated a 1.37% mean implant failure rate of immediately placed implants with socket shield technique at follow-up periods varying from 3 to 120 months, suggesting the importance of long-term follow-up.

Although this study yielded a 100% implant success rate, two external shield exposures were encountered. One of the exposure events in the maxillary central incisor submerged implant was due to a traumatic accident in that area leading to dislodgement of the resin bonded provisional bridge and traumatizing the overlying soft tissue. The other small exposure in the maxillary first premolar implant was due to a sharp corner of the shield in the mesiobuccal area that perforated the soft tissue. The two exposures were managed by just reducing the exposed part with a high-speed diamond bur, and both healed well with soft tissue coverage. Although more dimensional ridge shrinkage occurred for these two patients, good marginal bone stability and healthy peri-implant soft tissues were evident at the 12-month follow-up appointment. In addition, both patients were satisfied with the esthetic result and declined any connective tissue grafting procedure. Although two external exposure events were reported in this case series, the author considered that virtually one exposure had occurred since the second exposure was caused by a traumatic accident leading to soft tissue traumatization. Gluckman et al. [27] in a retrospective study of 128 socket-shield cases reported 16 occurrences of exposure. The authors advocated reducing the buccal shield to the level of the bone crest and creating a bevel in the coronal 2 mm of the shield to reduce the incidence of external and internal exposures [27].

The limitations of the present prospective case series study were the small sample size, the lack of a control group, the limited follow-up time, and its conduction in a single center rather than in multiple centers. In addition, the applied dimensional ridge change analysis might present some inaccuracy resulting from impression making, cast production, and the concentration on only the mid-root area for ridge width change analysis. Additionally, the contralateral tooth might represent a source of bias for this evaluation. It could be that a more accurate technique is to scan the ridge pre- and postimplantation and calculate the horizontal width changes with reverse engineering software of the two superimposed images, or to compare pre- and postoperative CBCT images.

More clinical studies involving multiple centers, a higher number of patients, and a longer follow-up period are required to correctly investigate the safety and accuracy of the socket shield technique in the medium- and long-term. In addition, this study lacked histological evidence from tooth extraction sockets with facial root fragment retention. This limitation can be overcome in further research using animal models.

## Conclusions

Within the limitations of this descriptive case series study, it can be concluded that the socket shield technique with maintaining the facial root segment could be a predictable minimally invasive option for cases demanding immediate implant placement. After 1 year of loading, all implants were successful and functional with excellent marginal bone stability and satisfactory esthetic results, while all patients revealed a wonderful satisfaction with the treatment outcome. Two patients presented with minor external shield exposure that was managed successfully without affecting the shield stability. However, it is still a sensitive technique that needs a learning curve and more robust evidence on its indications, suitable protocols, complications, and long-term functional and esthetic outcomes.

## Data Availability

The data that support the findings of this study are available from the corresponding author (R.S.), upon reasonable request. rola.shadid@aaup.edu
